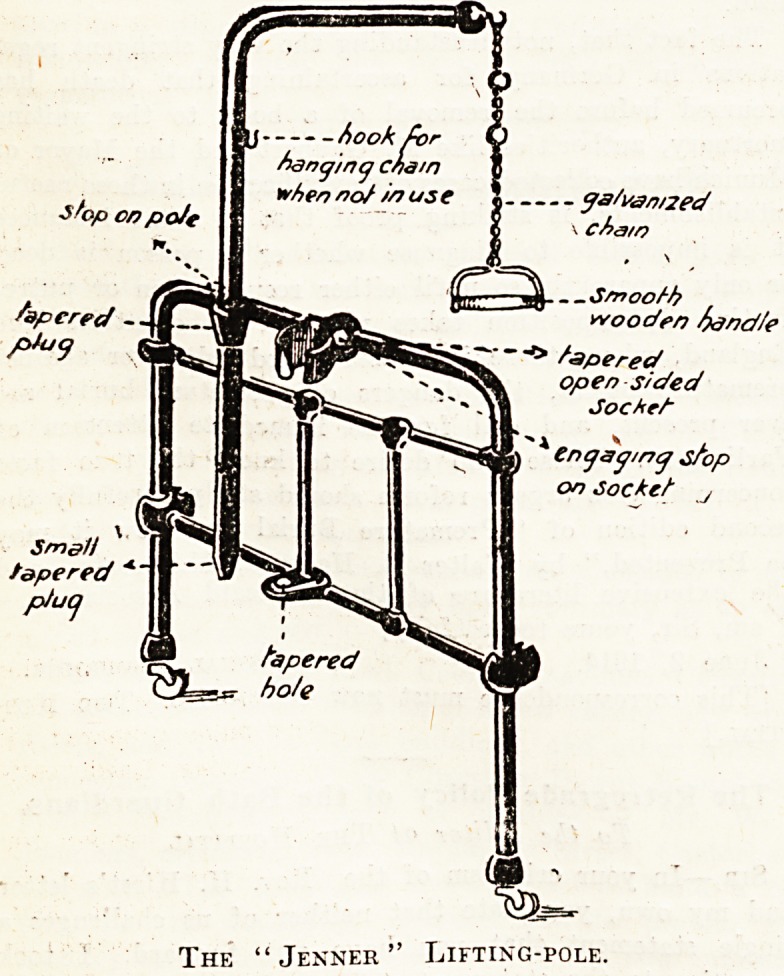# The "Jenner" Lifting Pole

**Published:** 1914-06-13

**Authors:** 


### The "Jenner" Lifting Pole.

In connection with our new competition, see page 3 of the cover, the following may prove interesting :—

The lifting-pole here described is in use at the Portsmouth Union Infirmary. The makers, Messrs. Fisher, Brown and Bayley, of Lionel Street, Birmingham, claim that it is very simple and inexpensive.

The pole itself, while not heavy, is exceptionally strong. The fitting on the bedstead end is in the shape of an open-sided tapered socket, and similarly on the lifting-pole is a tapered plug made to fit into the socket

The "Jenner" Lifting-pole.

on the bedstead end. At the bottom of the pole is another tapered plug, but smaller, which fits into a tapered hole. When it is desired to attach or detach the pole from the bedstead it is only necessary to lift it an inch. The pole then comes out through the open side of socket.

A good point claimed for this pole is that when it is attached to the bedstead the sockets and plugs, being tapered, make the fit perfectly rigid and secure, thus obviating any side play or rocking, its own weight ensuring perfect rigidity.

It is not fitted with any loose screws or nuts for tightening purposes, as is frequent in this class of hospital accessory, the tapered fittings being themselves self-tightening. It will, therefore, be seen that the pole cannot possibly become displaced. It is made with a stop which, when the pole is swung roughly, prevents it striking against the wall and damaging it. The handles may be of different shapes and material, and the chain is well galvanised to prevent rusting.

## Figures and Tables

**Figure f1:**